# Production of Size-Controlled Gold Nanoclusters for Vapor–Liquid–Solid Method

**DOI:** 10.3390/nano12050763

**Published:** 2022-02-24

**Authors:** Alam Saj, Shaikha Alketbi, Sumayya M. Ansari, Dalaver H. Anjum, Baker Mohammad, Haila M. Aldosari

**Affiliations:** 1 Department of Physics, United Arab Emirates University, Al Ain P.O. Box 15551, United Arab Emirates; alam.saj@outlook.com (A.S.); sumayya_a@uaeu.ac.ae (S.M.A.); 2 Department of Chemistry, United Arab Emirates University, Al Ain P.O. Box 15551, United Arab Emirates; 201504873@uaeu.ac.ae; 3 Department of Physics, Khalifa University, Abu Dhabi P.O. Box 127788, United Arab Emirates; dalaver.anjum@ku.ac.ae; 4 System on Chip (SoC) Center, Electrical Engineering and Computer Science, Khalifa University, Abu Dhabi P.O. Box 127788, United Arab Emirates; baker.mohammad@ku.ac.ae

**Keywords:** nanoclusters, sputtering, gold catalyst, nanowires, GeTe

## Abstract

This study demonstrated the deposition of size-controlled gold (Au) nanoclusters via direct-current magnetron sputtering and inert gas condensation techniques. The impact of different source parameters, namely, sputtering discharge power, inert gas flow rate, and aggregation length on Au nanoclusters’ size and yield was investigated. Au nanoclusters’ size and size uniformity were confirmed via transmission electron microscopy. In general, Au nanoclusters’ average diameter increased by increasing all source parameters, producing monodispersed nanoclusters of an average size range of 1.7 ± 0.1 nm to 9.1 ± 0.1 nm. Among all source parameters, inert gas flow rate exhibited a strong impact on nanoclusters’ average size, while sputtering discharge power showed great influence on Au nanoclusters’ yield. Results suggest that Au nanoclusters nucleate via a three-body collision mechanism and grow through a two-body collision mechanism, wherein the nanocluster embryos grow in size due to atomic condensation. Ultimately, the usefulness of the produced Au nanoclusters as catalysts for a vapor–liquid–solid technique was put to test to synthesize the phase change material germanium telluride nanowires.

## 1. Introduction

Gold (Au) nanoclusters had garnered the attention of researchers due to their unique physical and chemical properties, which could be primarily attributed to the high surface area-to-volume ratio [1]. Au nanoclusters proved to be of potential application in diverse fields including sensors [2], biomedicine [3], and catalysts [4]. Au in its bulk form does not depict any significant catalytic properties due to its high degree of ionization [5] and was paid less attention to be used as a catalyst as a result. It was only during the late 1970s that the utilization of Au as a catalyst for oxidation was explored, which suggested enhanced catalytic properties for Au nanoclusters of a smaller size range [5]. Further research conducted in the field in 1987 by Haruta et al. [6] demonstrated the catalytic properties of Au nanoclusters for the oxidation of carbon monoxide, which further proved its potential for catalytic applications and, hence, started being used extensively in this regard.

Of the different techniques available for metallic nanoclusters’ synthesis, direct-current (dc) magnetron sputtering has proven to be an effective way to attain metallic nanoclusters of a reduced size, uniform size distribution, and high purity. The dc magnetron sputtering technique, used in this study, has a few advantages over other techniques such as the simplicity in its operation, a highly controlled deposition rate, high-quality nanoclusters, synthesis capability of small and uniformly distributed nanocluster size, cost efficiency, and its ecofriendly nature [7].

Various researchers have used physical deposition means to produce size-controlled Au nanoclusters on various substrates/materials. For instance, Veith et al. investigated the catalytic activity of 2.1-nm and 1.7-nm Au nanoparticles deposited by dc magnetron sputtering on WO_3_ and activated C, respectively [8]. Au/WO_3_ and Au/C catalytic activities were tested by carbon monoxide oxidation and glycerol oxidation, respectively, exhibiting better performance compared to Au nanoparticles produced by chemical routes [8]. Baojia et al. fabricated 50–200-nm spherical Au nanoparticles by dc sputter Au thin film and subsequently annealed it at 900 °C for 20 min in a nitrogen environment [7]. Lee et al. synthesized 1-nm Au nanoparticles via inert gas condensation (IGC) and thermal evaporation at 1124 °C and 133 Pa [9]. Frankly, bimetallic Au nanoclusters garnered more attention than pure Au nanoclusters. Pérez-Tijerina et al. produced 1-, 3-, and 5-nm Pd/Au bimetallic nanoparticles by the IGC method from a 50:50 Au/Pd sputtering target [10]. Similarly, Mayoral et al. deposited 5-nm Pd/Au and Co/Au using IGC and successive alcoholic reduction [11]. Additionally, the impact of subsequent annealing on the nanoparticles revealed that Au nanoparticles are stable while Co was oxidized [11]. Nejm et al. deposited 4.8 ± 0.2-nm nanoparticles using dc sputtering and IGC methods for a hybrid organic memory device using a sputtering discharge power of 119 W [12].

Inspired by the aforementioned research and due to the lack of in-depth investigation of the impact of operational parameters on the synthesis of pure Au nanoclusters, we demonstrated a detailed investigation of the influence of various deposition source parameters, namely, dc magnetron sputtering discharge power (*P*), inert gas flow rate (*f*), and aggregation length (*L*) on Au nanoclusters’ average size and yield. Au nanoclusters obtained in this work were fabricated using dc magnetron sputtering combined with IGC methods. A discussion of Au nanoclusters’ formation and growth mechanism in terms of three-body and two-body collision was also presented. Ultimately, the produced Au nanoclusters were used as a catalyst for the vapor–liquid–solid (VLS) growth of the phase change material germanium telluride (GeTe) nanowires. Transmission electron microscopy (TEM) and scanning electron microscopy (SEM) were employed to characterize the Au nanoclusters and GeTe nanowires.

## 2. Materials and Methods

**Figure 1:**
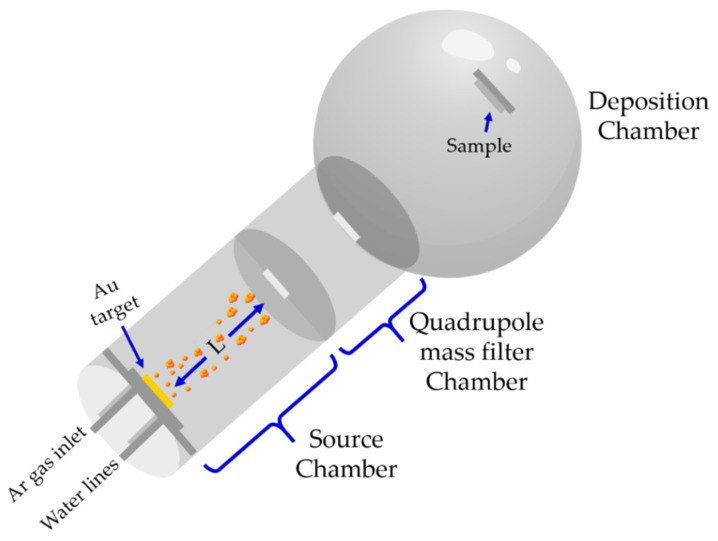
Schematic of the deposition system used in this study.

Au nanoclusters were deposited by the dc magnetron sputtering technique from a 2-inch Au sputtering target of 99.99% purity (Testbourne Ltd., Basingstoke, UK) using the Nanogen-50 deposition system (Mantis Deposition Ltd., Oxfordshire, UK). The system utilizes a combination of physical vapor deposition and IGC to produce nanoclusters from various materials. The system consists of three connected chambers in a raw, namely: source chamber, quadrupole mass filter (QMF) chamber, and deposition chamber, as illustrated in Figure 1 and described by Ayesh et al. [13]. Au nanoclusters are produced in the source chamber, size filtered via a quadrupole mass filter chamber, and deposited ultimately on the desired substrate in the deposition chamber.

Au nanoclusters’ generation in the source chamber was governed by three important parameters: magnetron sputtering discharge power (*P*), inert gas flow rate (*f*), and aggregation length (*L*). Argon (Ar) gas *f* was controlled by a MKS mass flow controller (MKS Instruments, Inc., Andover, MA) in the range of 50–90 sccm. *L* is defined as the region where the nucleation and growth of the nanoclusters take place. It is comprised of a dc power-supplied Au target (Cathode) and a linear actuator to vary the length of the aggregation region, which is the distance from the target surface to the source chamber exit nozzle. *L* was varied in the range of 40–90 mm. The source chamber walls are water-cooled to aid the condensation of the sputtered Au atoms.

On reaching the desired base pressure of 1.6 × 10^−6^ mbar, Ar gas was gradually purged into the system. With Ar atoms being very essential for the nucleation of Au nanocluster formation, studying the impact of *f* on the size and yield of the generated nanoclusters was one of the goals of this research. An increase in the Ar pressure within the chamber results in a change in the mean free path of the sputtered Au atoms, inducing more collisions with the Ar atoms and themselves, which lead to enhanced nucleation and growth [14]. After purging the system with Ar gas, the discharge power was supplied to the target, initiating the formation of plasma. Since it is a magnetron sputtering system, a powerful magnetic field is generated using a strong magnet, one pole of which is placed along the central axis of the target and the other in the form of a ring along the edges of the target to confine the secondary electron motion around the target [15]. These constrained electrons attract a greater number of inert gas ions to be attracted towards the target at a higher kinetic energy, thereby resulting in an increased rate of ion (Ar^+^) bombardments with the target, in turn ejecting a greater number of Au atoms [16]. The sputtered atoms then undergo condensation on colliding with the cooled aggregation tube walls [17] and Ar atoms within the aggregation region, resulting in Au embryos’ formation through a three-body collision mechanism, wherein two Au atoms and an Ar atom collide as follows.

Au + Au + Ar → Au―Au + Ar

The interaction between the Au atoms yields the formation of the Au dimer, which serves as the embryo for the nanocluster formation while the inert gas atom consumes the excess energy generated in the reaction [18]. The yield of the formed embryos increases with the density of the atomic vapor [13]. Afterward, the growth of the embryos into a nanocluster happens through a two-body collision mechanism through atomic condensation, where an embryo grows into a nanocluster by accepting the arriving adatom on its surface [19].

After nanoclusters’ formation, the nanoclusters are then transported through the QMF, which measures their size distribution. Measurement is recorded in the form of an intensity vs. diameter distribution curve. The QMF is comprised of four electrodes, which are supplied with a combination of dc (±U) and alternating-current voltage (±V cos(ωt)) potential difference. Therefore, when the charged nanoclusters pass between them, they are deflected based on their charge-to-mass ratio [20], which, in turn, leads to the filtration of the undesirably sized nanocluster, preventing it from reaching the deposition chamber. The U/V ratio represents the resolution of QMF and it was maintained at a constant value of 0.05 throughout the runs [13]. The produced nanoclusters are then passed through a grid that measures their ion flux, which is translated into a current (nA) using a picoammeter [13], which represents the number intensity of the nanoclusters that are being transported to the main chamber.

The effect of *P*, *f*, and *L* on the average diameter and yield of the produced Au nanoclusters was investigated by varying each one of them individually. A size distribution curve was recorded for each set of source parameters. Since the size distribution curve was observed to be a normal curve, the best estimate of the curve properties was confirmed by performing Gaussian fit for each curve to find the average diameter value and comparing the area under the curve, which represented the yield. The reported error values are the average standard deviations of the average diameter and yield found for each set of parameters. Au nanoclusters were deposited at room temperature on silicon (Si) substrates and TEM grids while rotating at 10 rpm to ensure uniform dispersion of nanoclusters. For all readings, the pressure was allowed to stabilize before recording the reading.

Transmission electron microscopy (TEM) analysis was carried out in the bright-field TEM mode by employing an aberration-corrected TEM of Titan 80-300 ST (Thermo Fisher Scientific Inc., Waltham, MA, USA). Au nanoclusters were deposited onto carbon-coated copper TEM grids. The imaging data were collected by setting the accelerating voltage to 300 kV. Several images at low as well as high magnifications were recorded. The high-resolution TEM images enabled imaging the crystal structure and size of Au nanoclusters.

For GeTe nanowires’ synthesis, Au nanoclusters were deposited on the Si substrate and annealed at 700 °C for 20 min to remove any organic contamination. The sample was then loaded into a 2-inch OFT-1200X tube furnace (MTI Corporation, Richmond, CA, USA) where 0.1 g of GeTe powder of 99.99% purity (Testbourne Ltd., Basingstoke, UK) was placed at the center of the tube below the Si substrate. After setting the temperature to 450 °C, the tube was then purged with Ar gas at a 120-sccm flow rate to serve as the carrier gas for the transportation of the vapor to the Au catalyst for the formation of GeTe nanowires. The working pressure was maintained at 75 torr during the synthesis, allowing the nanowires to grow for 4 h, followed by the sample being allowed to cool down to room temperature in ambient Ar. Au nanoclusters’ and GeTe nanowires’ morphology was also investigated using a Nova NanoSEM 650 scanning electron microscope (SEM) (Thermo Fisher Scientific Inc., Waltham, MA, USA).

## 3. Results and Discussion

**Figure 2:**
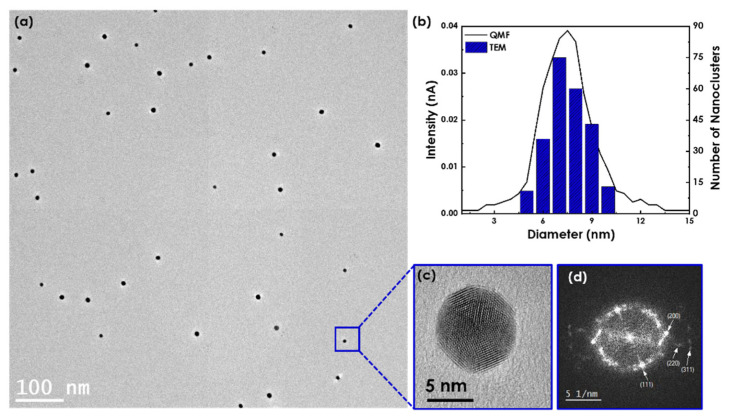
(**a**) Low-magnification, bright-field TEM image of the fabricated Au nanoclusters. (**b**) Size distributions of QCM and TEM. (**c**) High-magnification, bright-field TEM image of a single Au nanocluster and (**d**) its corresponding FFT.

A low-magnification, bright-field TEM image of size-controlled Au nanoclusters deposited at *P* = 12.2 W, *f* = 60 sccm, and *L* = 60 mm is shown in Figure 2a. Au nanoclusters’ size distribution found via QMF (solid line) was compared to the size found from TEM images (histograms) (in Figure 2b), showing great consistency and confirming the reliability of the QMF. Au nanoclusters produced with the aforementioned parameters’ values were found to have a narrow distribution with an average diameter of 6.46 ± 0.02 nm and a yield of 0.20 ± 0.01, as depicted in Figure 2b. A single Au nanocluster is presented in Figure 2c, with lattice fringes and atoms observable. Figure 2d depicts the corresponding fast Fourier transform (FFT), revealing the Au atoms’ cluster in the face-centered cubic (FCC) crystal structure. All Au nanoclusters were of spherical shape and crystalline with an FCC crystal structure.

**Figure 3:**
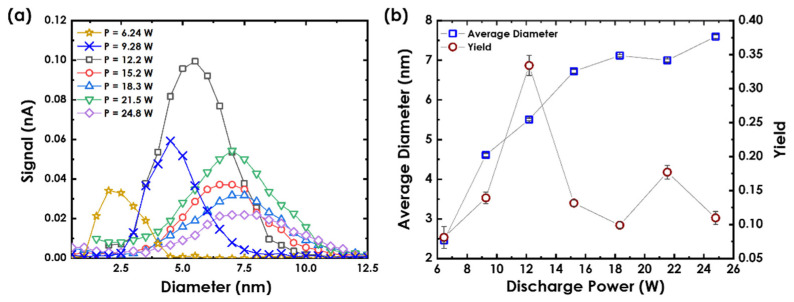
(**a**) Au nanoclusters’ size distributions as measured via the QMF at different *P*. (**b**) The dependence of Au nanoclusters’ average diameter and yield on *P* at *f* = 60 sccm and *L* = 60 mm.

The effect of *P* supplied to the target on Au nanoclusters’ average size and yield was investigated, while maintaining constant *f* and *L*. Figure 3a illustrates the size distributions of Au nanoclusters at *f* = 60 sccm and *L* = 60 mm for different *P* values. Since *P* is directly linked to the rate at which Au atoms are ejected from the target into the vapor within the aggregation region, it is representative of the amount of Au atoms available within the chamber to initiate the formation of the nanoclusters. Using *P*, less than 6.2 W did not produce enough self-bias on the Au target to generate Au nanoclusters [13]. Au nanoclusters were only detectable after using 6.2 W, producing 2.5 ± 0.1-nm Au nanoclusters. The nanoclusters’ average diameter was defined as the peak of the Gaussian fitting curve of the size distributions in Figure 3a, which is projected as blue square data points in Figure 3b. The peak of the nanocluster size distribution exhibited a shift toward larger average diameters (towards the right) with increasing the value of *P*. This can be attributed to the increased amount of ejected Au atoms from the target with increasing *P*. Since this increased the Ar ions’ bombardment energy, it resulted in the formation of larger nanoclusters. For *P* > 15.2 W, the increment of Au nanoclusters’ size with *P* was slight and saturated at around ~7 nm. On the other hand, the nanocluster yield represented by the area under each size distribution in Figure 3a is converted into red circle data points in Figure 3b. The Au nanoclusters’ yield increased to its maximum value of 0.33 ± 0.01 at 12.2 W and then progressively decreased. This can be ascribed to the Au nanoclusters’ nucleation mechanism.

**Figure 4:**
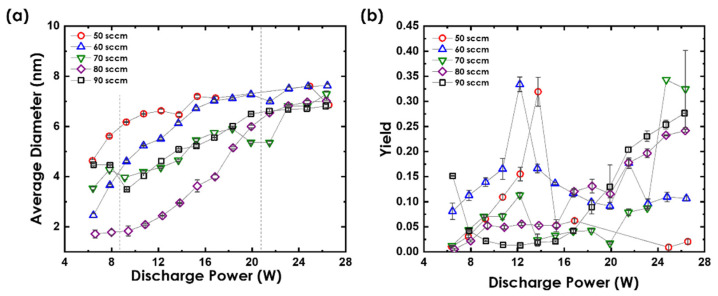
Impact of *P* on Au nanoclusters’ (**a**) average diameter and (**b**) yield presented for various *f* at *L* = 60 mm.

Having the data for one *f* could limit the precision of the analysis. Therefore, the argon gas that was purged into the system was varied between 0 and 90 sccm for its effect to be better understood. Ar atoms are very essential for determining the nucleation mechanism of Au nanoclusters’ formation within the source chamber. An increase in the Ar pressure within the chamber results in a change in the mean free path of the sputtered Au atoms, inducing more collisions with the Ar atoms and themselves, which lead to enhanced nucleation and growth [14]. Hence, the effect of *P* on an Au nanocluster’s average diameter and yield was further studied for different *f,* as illustrated in Figure 4a,b, respectively. It should be noted that using *f* < 50 sccm would either produce Au nanoclusters that are not detectable or generate scattered data points that do not follow any trend due to unstable plasma. Hence, we confined our analysis of *f* from 50 to 90 sccm. Overall, the average diameter of Au nanoclusters shifts toward larger sizes with increasing *P*, as the density of the Au atomic vapor increases with *P*. This increases the probability of atomic condensation, producing larger nanocluster sizes. The saturation of an average diameter happens at *P* values > 19.9 W for all *f* values. The produced Au nanoclusters showed a confined nanoclusters’ size range from 1.7 ± 0.2 nm to 7.6 ± 0.1 nm.

As *f* increased from 50 sccm to 80 sccm for the same *P* values, the average diameter decreased, which was attributed to the reduction in the mean free path of collision. As *f* increased, the abundance of Ar molecules in the source chamber caused an increase in the number of collisions between the Au clusters and Ar molecules, making nucleation through a three-body collision the dominant phenomena in the source chamber. This deceased the rate of nanocluster aggregation, producing Au nanoclusters of reduced size. For *f* = 90 sccm, the average size increased again for *P* < 19.9 W but showed a similar trend at high power values. At *f* = 90 sccm and low *P*, the two-body collision mechanism dominated the growth, producing a smaller amount of larger Au nanoclusters. The minimum Au nanocluster diameter recorded was 1.7 ± 0.2 nm for *f* = 80 sccm at *P* = 6.6 W, while the largest particle size was observed to be 7.6 ± 0.1 nm at *P* = 26.4 W and *f* = 60 sccm, both at *L* = 60 mm. Nejm et al. produced 4.8 ± 0.2-nm Au nanoclusters using the same system using *P* = 119 W, *f* = 80 sccm, and *L* = 60 mm [12]. One can see from Figure 4a that using the same conditions but a lower power of 18.3 W produced nanoclusters with the same size, reflecting the importance of the optimization of source parameters to achieve full control of Au nanoclusters’ size.

Figure 4b shows that nanoclusters’ yield depends on *P* and *f*. For *f* < 80 sccm, the yield increased as *P* increased to attain a maximum value and then decreased. For *f* ≥ 80 sccm, the nanoclusters’ yield increased gradually over the range of *P* values to saturate at ~0.25, which could have been due to the reduction in the nanoclusters’ size. The yield was observed to be decreasing with an increasing nanocluster size (Figure 4a,b). This could be because, as more clusters merged to form larger clusters, the increase in nanoclusters’ size happened at the expense of their number. Another interesting observation to be made was that for every value of *f*, the yield peaked at a point where a certain balance was attained between the *f* and *P*. For instance, at *f* = 60 sccm, the peak of the yield occurred at *P* = 12.2 W, while at *f* = 70 sccm, the peak shifted towards the right, occurring at *P* = 13.7 W. This explains a direct correlation between the two parameters. Since Ar atoms and sputtered Au atoms are both essential for the generation of nanoclusters through a three-body collision mechanism, an increase in the ratio of any one of them could significantly decrease the rate of nucleation, which is reflected the number of nanoclusters generated. Additionally, the pressure difference between the aggregation region and the region outside the source exit is one factor that affects the removal of the nanoclusters from the aggregation region. Therefore, exceeding the Ar gas pressure within the aggregation chamber over a certain threshold value could lead to immediate removal of the atoms from the region without letting enough time for nucleation and growth [21]. Therefore, maintaining a moderate *f* is very crucial for the controlled deposition of the Au nanoclusters. The maximum values of the yield were obtained when the *f* and *P* were maintained at an intermediate level for *f* = 50 sccm to *f* = 70 sccm, while the yield increased with *P* at higher *f* values. This explained the requirement of the two parameters to be maintained at the same proportion to obtain a high yield.

The amount of sputtered material and the stability of the plasma were controlled by *P*. Using low values would be insufficient to sputter a detectable amount of Au nanoclusters from the target, whereas using high values would affect the stability of the plasma and can utterly damage the Au target as it is a ductile material and using high power would drill on the target and permanently damage it. Hence, a power of 12.2 W was used for the rest of the work as it produced the maximum yield without affecting the integrity of the Au sputtering target.

**Figure 5:**
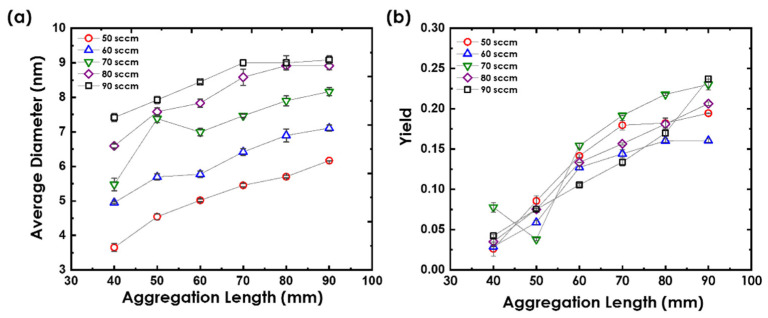
Au nanoclusters’ (**a**) average diameter and (**b**) yield dependence on *L* shown for various *f* at *P* = 12.2 W.

Another parameter that significantly affected the size and yield of Au nanoclusters was *L*, which is defined as the distance between the source target and the source chamber’s exit nozzle. It also determines the time duration for which the generated nanoclusters remain within the aggregation region, which is again proportional to the nanoclusters’ size [13]. To study the dependence of Au nanoclusters on *L*, *P* was fixed at 12.2 W and *L* was varied at different values of *f* between 50 sccm and 90 sccm, as depicted in Figure 5a.

As *L* increased, Au nanoclusters’ average diameter gradually increased, which can be explained by the nanoclusters staying longer in the source chamber, which increased the time of nucleation and growth via atomic condensation through a two-body collision mechanism, producing larger nanoclusters. Therefore, the longer they remain within the aggregation region, the greater is the chance for them to interact with each other, resulting in an increased nanocluster size distribution. Moreover, for the same *L* value, the average diameter of Au nanoclusters remarkably increased with increasing *f*, confirming that Au nanoclusters grow via a two-body collision mechanism.

The highest nanoclusters’ size was generated at the highest values of *L* and *f* (90 mm and 90 sccm) while the lowest was produced at the lowest values of *L* and *f* (40 mm and 50 sccm). The average diameter was observed to be stabilizing after *L* = 70 mm for *f* ≥ 80 sccm. Varying *f* could be used as an effective strategy to vary the nanocluster size, particularly if we are to achieve a significant change in the diameter.

On the other hand, Au nanoclusters’ yield slightly increased with *L* for all *f* values, as illustrated in Figure 5b. Moreover, it did not show any dependence on *f*. The increase in Au nanoclusters’ yield was more profound for larger *L* values, wherein the size of the Au nanoclusters slightly increased or remained unchanged, which could be explained to be happening as a result of the production of more nanoclusters at the expense of enlarging the nanocluster size.

## 4. Application

**Figure 6:**
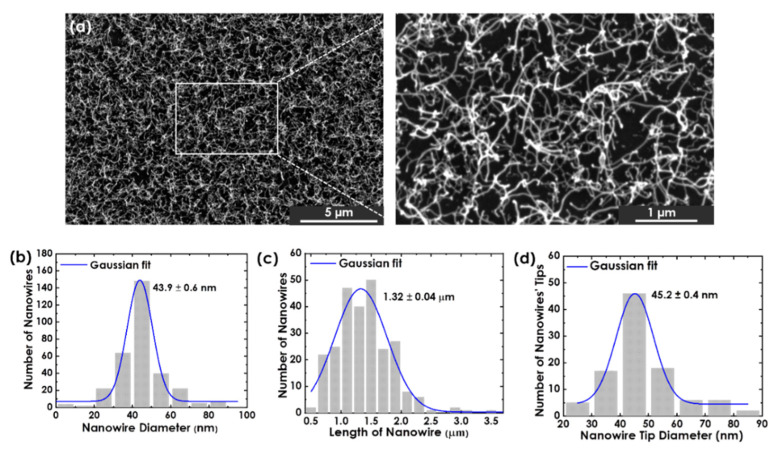
(**a**) SEM micrographs of GeTe nanowires, forest grown via the VLS method using 5.80 ± 0.10-nm Au nanoclusters with a yield of 0.10 ± 0.0 deposited for 1 h. Histogram of GeTe nanowires’ (**b**) average diameter and (**c**) length. (**d**) Histogram of the size of Au nanoclusters attached to synthesized GeTe nanowires as a result of the VLS method.

To demonstrate the utilization of Au nanoclusters produced in this work as a catalyst for the VLS technique, GeTe nanowires were synthesized using 5.8 ± 0.1-nm Au nanoclusters as a catalyst, as shown in Figure 6a. A spherical Au tip was observed at the end of all nanowires, which is a typical feature for the VLS technique. GeTe nanowires of 43.9 ± 0.6-nm average diameter and 1.32 ± 0.04-µm length were produced, as shown in Figure 6b,c, respectively. Although the as-deposited Au nanoclusters’ size used in the GeTe nanowires’ synthesis was 5.80 ± 0.10 nm, the average size found for the Au tips at the edge of the GeTe nanowires was 45.2 ± 0.4 nm, as resolved via SEM (Figure 6d).

**Figure 7:**
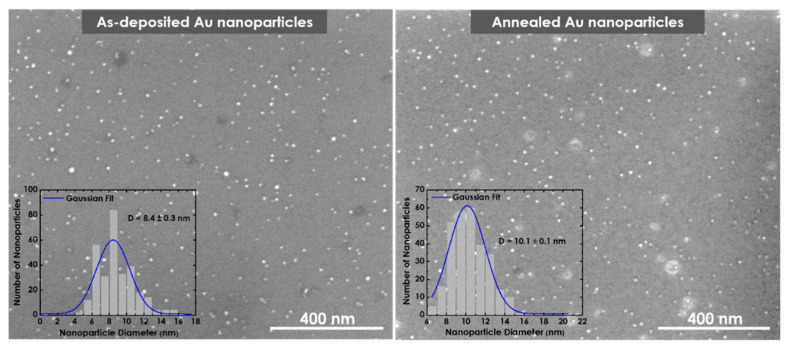
SEM micrographs of Au nanoclusters before and after annealing at 700 °C for 20 min.

It should be mentioned that prior to GeTe nanowires’ synthesis, Au nanoclusters were annealed at 700 °C for 20 min to remove any organic contamination. Figure 7 shows Au nanoclusters deposited using *P* = 17.7 W, *f* = 70 sccm, and *L* = 75 mm, before and after annealing; their sizes only increased slightly after annealing due possible agglomeration of nearby nanoclusters [7], indicating that the increase in the Au tip size was not because of annealing. We ascribed one possible reason for such a large increase in the nanocluster size to the lateral growth of GeTe nanowires as more nanoclusters attached themselves to the initial 5.8-nm nanocluster. This explains the wide dispersion of the attached Au tip diameter of the produced GeTe nanowires, as shown in Figure 6d. It should be mentioned that this phenomenon is currently being studied for a better explanation.

## 5. Conclusions

In summary, we reported the production of size-controlled Au nanoclusters synthesized using inert gas condensation and dc magnetron sputtering. The impact of magnetron discharge power (*P*), inert gas flow rate (*f*), and aggregation length (*L*) on the diameter and yield of Au nanoclusters was thoroughly investigated. To generate small < 5-nm Au nanoclusters, low *P*, small *L*, and large *f* were needed, and vice versa to produce larger nanoclusters. A good agreement was achieved between the size distributions of Au nanoclusters obtained from the quadrupole mass filter and the transmission electron microscope images. Optimization of source parameters produced monodispersed nanoclusters of an average size range of 1.7 ± 0.2 nm to 9.1 ± 0.1 nm. Results indicated that Au embryos grew into large nanoclusters via a two-body collision mechanism. Ultimately, the produced Au nanoclusters were used to synthesize very thin and stand-alone phase change material nanowires via the vapor–liquid–solid method for its envisioned applications.

## Data Availability

The data that support the findings of this study are available from the corresponding author upon request.
